# Influences of Extraction Methods on Physicochemical and Functional Characteristics of Three New Bulbil Starches from *Dioscorea opposita Thunb*. cv. Tiegun

**DOI:** 10.3390/molecules24122232

**Published:** 2019-06-14

**Authors:** Pengzhan Zhang, Li Wang, Yanyan Qian, Xuguang Wang, Shaoning Zhang, Jiping Chang, Yuan Ruan, Bingji Ma

**Affiliations:** 1 Department of Traditional Chinese Medicine, College of Agronomy, Henan Agricultural University, Zhengzhou 450001, China; zhangpz0625@163.com (P.Z.); wanglihuina@163.com (L.W.); qian18838922613@163.com (Y.Q.); cjpmba@163.com (J.C.); ruanyuanmbj@163.com (Y.R.); 2 Baiyunmugang Biological Technology Company, Dengfeng 452471, China; wangxuguanghn@163.com (X.W.); zsn1st@163.com (S.Z.)

**Keywords:** *Dioscoreae opposita* Thunb. cv. Tiegun, Starch, Different extraction methods, Physicochemical and functional properties

## Abstract

Starches from the bulbils of *Dioscoreae opposita* Thunb. cv. Tiegun were isolated by aqueous steeping (SBS), enzyme extraction (EBS), and alkaline extraction (ABS) methods, respectively. The physicochemical, mineral composition, thermal and morphological characteristics of these starches were investigated. The starch granules were oval, spherical and kidney-shaped and its crystal type is a mixture of A-type and B-type patterns. The starches having larger average granule size showed more amylose and phosphorus contents than those with smaller average granule size. Differential scanning calorimetry (DSC) showed that the SBS had an endothermic transition ranging from 65.8 °C to 76.3 °C with an enthalpy of 2.0 J/g. The endothermic transitions of ABS and EBS showed the regions of 67.9 °C to 73.0 °C, and 66.8 °C to 82.0 °C, respectively. The gelationization enthalpies of ABS and EBS were 13.8 and 11.5 J/g, respectively. Additionally, ABS presented greater clarity in comparison with EBS and SBS. Pasting properties indicated that ABS had the highest peak viscosity, breakdown, but SBS had the lowest trough, final viscosity, setback, and pasting temperature. Generally, ABS and EBS could be used as food thickener or frozen food additives. SBS and EBS were potential technological alternatives in quality preservation of frozen starch-based products and other industrial applications.

## 1. Introduction

*Dioscoreae opposita* Thunb of varieties of cultivars are planted in China, particularly in Henan Province, for medical and food purposes. Among them, the most sought is *Dioscoreae opposita* Thunb. cv. Tiegun, which is commonly named *“Huai Shan Yao*” in China. This cultivar is edible and has been routinely used to treat diarrhoea, diabetes and asthma for over 2000 years (*Chinese Pharmacopoeia*, 2015 edition) [1]. According to historic text “Ben Cao Gang Mu” written by Li Shi-zhen in the Ming dynasty (1368–1644 A. D.), this species was already cultivated during medieval times in China for the treatment of various illnesses. 

Starch, an important plant polysaccharide, is the most abundant carbohydrate and the main ingredient in the *Dioscoreae opposita* Thunb. cv. Tiegun. Recently, it was reported that the above- and under-ground parts of the same edible plants appeared to produce starches with different properties, which could be used to serve different purposes in the food and non-food industries [2]. However, during harvest, the bulbils of *Dioscoreae opposita* Thunb. cv. Tiegun were left behind and discarded in the field, otherwise representing an excellent natural resource of starch. Starches from bulbils possess attractive physicochemical properties; starches generally are non-toxic and biodegradable, therefore, they have been applied in food, pharmaceutical, and polymer industries. 

Starches are extracted from plant sources, using alkaline, acid, or enzymatic methods. Different extraction methods often result in differences in the yield and quality of the extracted starch. Maniglia and Tapia-Blacido [3] reported that the alkaline method provided higher extraction yield and purer starch from babassu mesocarp. In contrast to the enzymatic method, the alkaline extraction method conferred superior starch extraction yield from chestnut (*Castanea sativa* Mill.) and acorn (*Quercus suber*) [4]. Four different extraction methods (water, pectinase, sodium hydroxide and oxalic acid/ammonium oxalate) were evaluated and compared for the extraction of starch from *Dioscorea alata* [5], with the oxalic acid/ammonium oxalate method having the best starch recovery and granular variation, but altered rheological behavior. Thus, functional characterizations of solubility, swelling, and water absorption capacity are important for the diverse applications of starches. The current work has evaluated effects of different isolation methods (aqueous steeping, alkaline steeping and enzyme steeping) on the physicochemical, functional, and structural properties of bulbils starch of *Dioscoreae opposita* Thunb. cv. Tiegun.

## 2. Results and Discussion

### 2.1. Starch Yield and Chemical Composition

Three different extraction methods were applied to extract starch from fresh bulbils of *Disoscorea opposite* Thunb. cv. Tiegun, and their starch extract yields and composition were compared. As shown in Table 1, the starch extraction yields were 29.9%, 25.5% and 23.8% for the aqueous steeping method, enzyme extraction method and alkaline extraction method, respectively. The starch yield of SBS was significantly higher than that of EBS and ABS. Meanwhile, EBS yield was significantly higher than that of ABS, which could be attributed to the larger disruption of cell walls, and thus, the more efficient release of starch, comparing to that of ABS [6]. However, for all three different extraction methods, starch remained the dominant product ranging from 88.9% to 91.7% of the total extracted materials. The moisture content, which was influenced by the crystalline structure of the starch granule, ranged from 5.5% to 8.1% in the bulbil starches [7]. Both protein and ash contents were very low in extracted materials. The lipid content was found to be in the range of 0.71% to 0.84%, which was higher than that of potato and cassava starches, and similar to that of oat starch [8,9].

It is also worth pointing out that the protein contents of 0.01% in the extracted starches reported here are much lower than the FDA limit of corn starch of 0.4% [10]. The amylose contents in the starches from these different extraction methods differed slightly, but were higher than those in the reported data [11]. The amylose content in starch has been reported to influence the physicochemical properties and reactivity of starches. Starches with high amylose content are thought to be suitable for making low water-permeable biomembranes, food additives, or coating materials in food and pharmaceutical industries because of their excellent film-forming properties [12]. In addition, high-amylose starches have potential health benefits. They are positively correlated to a high content of resistant starch, which has been related to lowering glycaemic and insulin responses as well as reducing the risk for developing type II diabetes, obesity and cardiovascular diseases [12]. The amylose proportion also affected the starch paste viscosity, and low amylose content plays an important role in starch physicochemical and functional properties. The retrogradation phenomenon was delayed in the low-amylose starch [13]. The mineral composition and content of three bulbils starches contained a relatively high 127–174 mg/kg of phosphorous, 39.4–96.08 mg/kg of magnesium, 255–464 mg/kg of calcium, and 10.49–55.59 mg/kg of zinc. While three bulbils starches contained relatively low 1.08–1.13 mg/kg of boron, 0.7–1.32 mg/kg of cuprum, and 0.04–0.21 mg/kg of selenium. The potassium was not detected in three samples and the iron was only detected in EBS. The sodium contents of SBS (30.39 mg/kg) and EBS (58.06 mg/kg) were significantly higher than that of ABS (4.08 mg/kg). These minerals could all be associated with the internal structure of the starch. Phosphorus is one of the non-carbohydrate constituents present in the starches, which significantly affects the functional properties of the starches. There was no statistical difference (*p* < 0.05) between EBS and ABS, which indicated that the effect of enzyme extraction and alkaline extraction methods on the phosphorus content of starches was not significant. Maybe small starch particles are rich in phosphorus, but they were removed in the pupled and multiple washing procedures. Therefore, the phosphorus content in SBS were lower than those of the other samples. The phosphorus contents of the bulbil starches were similar to that of *Dioscorea septemloba* Thunb starch (0.011–0.015%) and lower than that of *Dioscorea trifida* starch (0.03–0.07%) [14]. Sodium, calcium, and magnesium levels tended to be higher when the granules were smaller, which was consistent with other reports. Additionally, differences in the mineral composition and content in these starches could also affect pasting viscosity and properties. Also, the calcium effect on the pasting properties could be stronger because of the excessively high calcium content [15].

### 2.2. X-ray Diffraction

**Figure 1:**
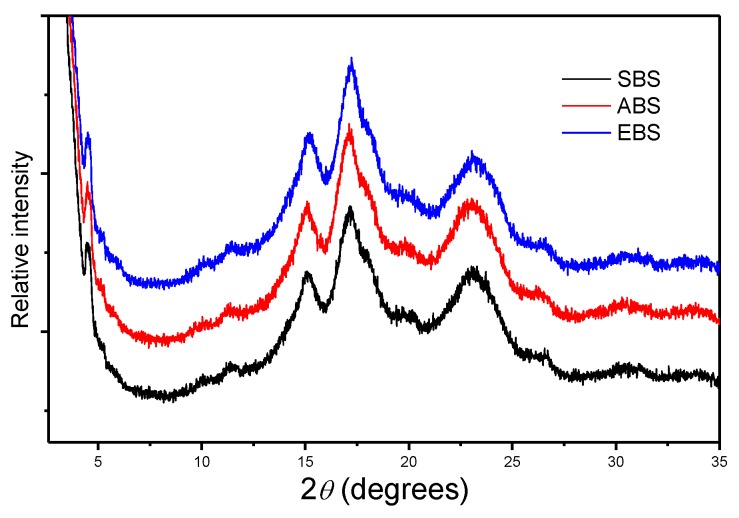
X-ray diffraction patterns of SBS, EBS and ABS.

X-ray diffraction (XRD) patterns of starches obtained by three methods from bulbils of *Dioscorea opposite* Thunb. cv. Tiegun are presented in Figure 1. The identification of a crystalline phase of any sample can be done by using a powder diffraction file. Each one of the amylose peaks was directly identified to produce diffraction using the powder diffraction file No. 43-1858 [16,17]. SBS, ABS and EBS have similar peaks at approximately 15.019° (111), 17.143° (301) and 23.007° (511) in a 2*θ* scale, which indicated that these starches have both monoclinic and hexagonal structures [18]. The type-A and type-B correspond to the monoclinic structure and hexagonal structure of starches, respectively [18,19]. All the diffraction peaks were similar to those of bulbils starches obtained by Zhang et al. and Zhou et al. [11,20]. The slight differences of diffraction peak intensities among EBS, ABS and SBS could be attributed to the changes in the monoclinic or hexagonal structures (size, interstitial atoms, etc). In addition, the crystalline structure of amylose and amylopectin as well as the lamellae formation in these bulbils starches are still an open problem that requires more calculations and studies [21].

### 2.3. FT-IR of Starch

**Figure 2:**
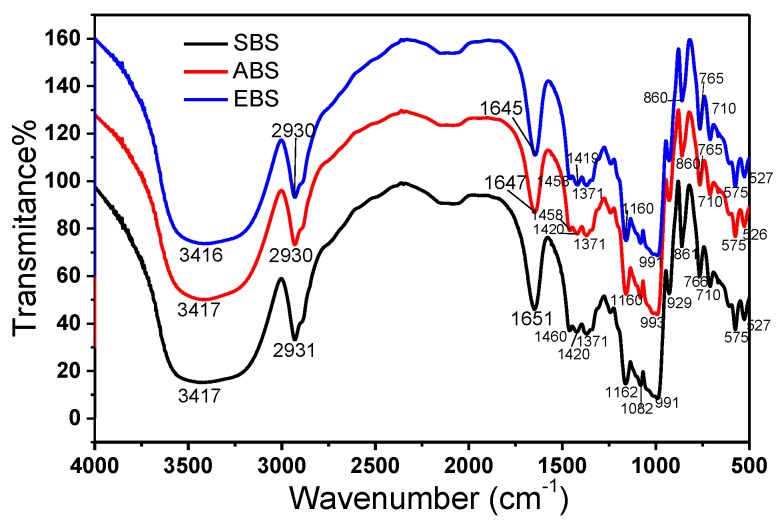
Fourier transform infrared spectra of SBS, EBS and ABS.

In order to identify the main functional groups in the above three starch samples, FT-IR analysis of the samples were conducted. As shown in Figure 2, the three FT-IR spectrum were similar to each other, indicating all three starch samples contain similar functional groups. 

The broad band approximately at 3416 cm^−1^ is due to complex stretching vibration of the intra- and intermolecular or free OH groups. The absorbance at 2930 cm^−1^ is due to the vibrational stretch of the C–H bond of the glucose structural component. The absorption at 1646 cm^−1^ probably arose from the scissor vibrations of OH of the bound water molecules [22]. Absorbance at 1460 cm^−1^ and 1420 cm^−1^ is characteristic of the C–H bond foldings. Consistent with the extremely low protein content in the extracted starches (Table 1), the above FT-IR spectra are absent of the signature band at 1560 cm^−1^, which is characteristic for amino group deformations [23]. 

The main absorbance by starch molecules could be observed in the region between 1200 cm^−1^ and 1000 cm^−1^, which largely encompass C–O, C–C and C–O–H stretching and C–O–H bending [7]. Typically, all three sample molecules showed absorption at 1162 cm^−1^ (coupling modes of C–C and C–O stretching), 1082 cm^−1^ (stretching vibration of C–O–H from glycosidic bonds) [9], and 991 cm^−1^ (C–O stretch and C–O–C vibration in the anhydroglucose ring). The absorption at 929 cm^−1^ is probably due to the skeletal mode vibration of α-1, 4-glycosidic linkage, which is known to be sensitive to water content and the characteristic index of hydrophilicity of starches [24]. While the peak at 766 cm^−1^ is attributable to C–C stretching, the bands at 710, 575 and 527 cm^−1^ probably arose from the skeletal modes of the pyranose ring in the glucose unit of starches [25].

The bands at 1047 and 1022 cm^−1^ were reported to be sensitive to the amount of crystalline (ordered structure) and amorphous (disordered structure) starch, respectively. The ratio of absorbance at 1047 and 1022 cm^−1^ is used to define the conformation of starch, and a high ratio means a large crystalline region of starch [11]. The ratios for all three starches, 1.23 for the alkaline extraction method, 1.21 for enzymatic method and 1.21 for the aqueous steeping extraction method, were similar with no significant difference, indicating all three extraction methods produced starches with similar conformations.

### 2.4. Scanning Electron Microscopy

**Figure 3:**
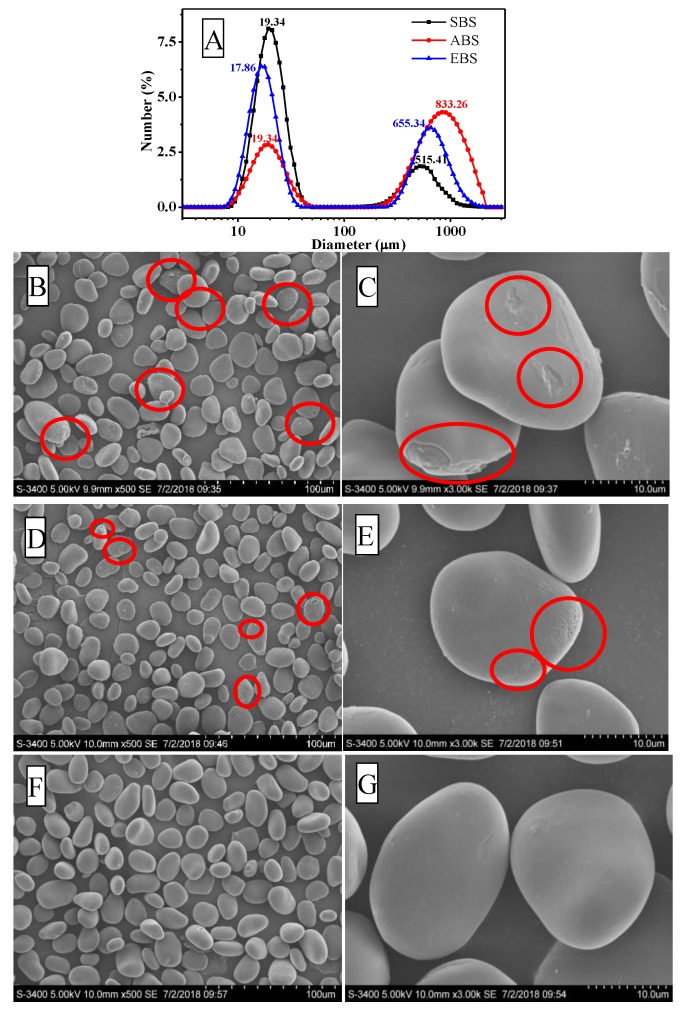
The starch granule size distribution of starches obtained by aqueous steeping, enzyme extraction, and alkaline extraction (**A**). The results are expressed by the relative percentage (*y*-axis) of particles of a diameter ranging from 0.02 to 2000 μm (*x*-axis, logarithmic scale). Scanning electron micrographs of SBS (**B**,**C**), ABS (**D**,**E**), and EBS (**F**,**G**). Red circles:Small visible cracks in the surface of starches.

Scanning electron microscopy analyses were performed to examine the surface characteristics and shape of starches obtained by the three extraction methods. As shown in Figure 3, the starch granules of *Dioscoreae opposita* Thunb. cv. Tiegun bulbils were oval, spherical and kidney-shaped. The surface of EBS granules appeared to be smooth, relatively uniform and dense with no apparent fissure, which was attributed to the gentle extraction condition. In contrast, the starches obtained by SBS and ABS methods appeared to have small visible cracks and showed surface roughness, which might be a result of re-association within the granule [26]. The alkaline extraction process could cause disruption of starch granules, de-polymerization and cross-linking of starch granules. The different surface morphology of the starch granules might lead to functional alteration of starch [27]. Each starch granule size distribution shows a bimodal pattern in Figure 3A. The first peak was observed at a size of 19.34 μm ± 1.02 in SBS, which was similar to that of ABS (19.34 μm ± 0.99), while the second peaks were at a size of 515.41 μm ± 0.82 (SBS) and 833.26 μm ± 1.04 (ABS), respectively. The first peak was obtained at a size of 17.86 μm ± 0.03 in EBS, while the second peak was at a size of 655.34 μm ± 0.32 (EBS). There were no significant differences among the first peaks of starches obtained by the three different methods [28].

### 2.5. Thermo-Gravimetric Analysis

The DSC thermograms of samples showed typical glass transitions of a semi-crystalline biopolymer. The transition temperatures (onset [*T_o_*], peak [*T_p_*], and conclusion [*T_c_*]), gelatinization temperature range (*T_c_−T_o_*), and gelatinization enthalpy (DH) of three different starches are exhibited in Table 2. 

DSC showed that SBS had an endothermic transition ranging from 65.8 °C to 76.3 °C with an enthalpy of 2.0 J/g. The endothermic transitions of ABS and EBS showed the regions of 67.9 °C to 73.0 °C, and 66.8 °C to 82.0 °C, respectively. The gelationization enthalpies of ABS and EBS were 13.8 and 11.5 J/g, respectively. The gelatinization temperature for bulbils starches of *Dioscoreae opposita* Thunb. cv. Tiegun is higher than that reported for corn (62.4 °C, 66.3 °C and 72.9 °C) [29] and lower than those of starches from rhizome (73.6 °C, 83.1 °C and 88.6 °C) and bulbil (71.7 °C, 81.1 °C and 89.2 °C) of Chinese yam [20]. The differences in gelatinization temperatures in starches from different varieties may be attributed to the various factors including the composition of the starch granule (the rate of amylose and amylopectin), the crystalline/amorphous structure, starch granular shape and size, the molecular structure of amylopectin, and the content of other components [30]. The higher temperature of gelatinization indicated the higher stability of the starch crystallites, which means that more heating is required to swell the granules [31]. Hence, the stability of ABS was higher than the other two starches. A similar result was reported that amaranth starch obtained by the strong-alkaline method showed a higher gelatinization temperature (70.6 °C) than the starch obtained by the low alkaline-protease method at 65.5 °C [32].

The gelatinization is indicative of the amount of energy to disrupt double helices and causes the loss of molecular order. The ordered structures may be formed by long chains. The greater the amount of ordered structures required, the more thermal energy to break the crystalline order [33]. Gelatinization enthalpy was significantly different in these three samples (*p* < 0.05). Among them, SBS had the lowest enthalpy of gelatinization (2.0 J/g) while ABS had the highest enthalpy (176.4 ± 0.23 J/g). High gelatinization enthalpy of ABS might be due to its high crystallinity, high proportion of long chains and great average chain length [34]. In addition, the low ΔT value (5.1 °C) of ABS indicated the high homogeneity and purity of starch. 

### 2.6. Swelling Power and Solubility

**Figure 4:**
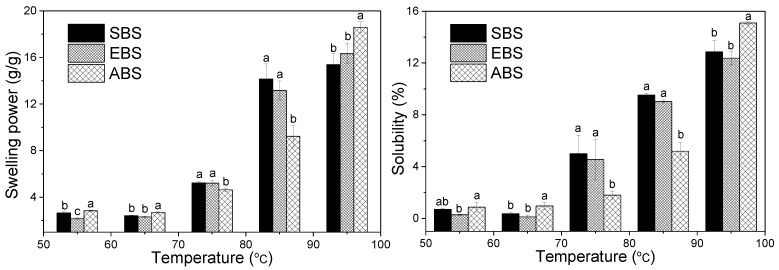
Swelling powers and solubilities of SBS, EBS and ABS (Data were reported in means±SD (*n* = 3); values in the same row with different superscripts are significantly different (*p* ˂ 0.05).

Figure 4 shows the swelling behavior and solubility of SBS, EBS and ABS at different temperatures (55 °C, 65 °C, 75 °C, 85 °C, and 95 °C). The solubility and swelling power were affected by the contents of amylose and amylopectin. Amylopectin contributes to the swelling of starch granules, in contrast, amylose, lipids and proteins inhibit the swelling of starch [35]. Hence, the differences in the structural features that influenced the swelling power would ultimately influence the starch pasting profile. Due to the crystal structure, the starch could be suspended and not easily dissolved in cool water. As the temperature increased, the starch solubility increased because the amylopectin and amylose started to dissociate and dissolve in water. The insoluble starch granules started to swell due to the hydration [36]. The ability of starches to swell in excess water and their solubility also differed (Figure 4). They could be used to assess the extent of interaction between starch chains in the crystalline and amorphous domains [37]. 

The swelling powers were similar as the temperature increased from 55 °C to 65 °C, indicating that starch granules did not swell at temperature below 65 °C, and this is consistent with the DSC result. Comparing with the values obtained at 65 °C, once the temperature of 95 °C was reached, the swelling powers of starches increased by approximately 13.0 (g/g), 14.0 (g/g) and 15.9 (g/g) for SBS, EBS and ABS. The effect of temperature on starch swelling power is attributed to the greater vibration of the molecules at a higher temperature, causing the combination of starch with the water molecule to form hydrogen bonds. The results illustrated that starches swelled quickly from 75 °C to 85 °C, and the swelling power of ABS was lower than that of SBS and EBS. Additionally, the swelling powers of SBS, EBS and ABS at 95 °C were lower than normal potato (1159 g/g) and normal corn (22 g/g), but higher than high amylose corn (6.3 g/g) [38]. The low swelling power of starches might be because of the presence of a large number of crystallites formed by the association of long amylopectin chains [39].

The results showed that the solubilities of EBS, ABS and SBS increased slightly with the increase in temperature from 55 °C to 65 °C, whereas they increased significantly from 0.4% to 12.9%, 0.1% to 12.4%, 1.0% to 15.1% for SBS, EBS and ABS in the range of 65 °C to 95 °C, respectively. Since the solubility of the sample could be affected by the amylose content, amylose-rich starch has lower solubility even after long heating periods [40]. The solubility of EBS and ABS did not differ significantly, indicating that the alkaline and enzyme extraction methods did not greatly affect the sample solubility. In addition, the solubility was related to the swelling power, due to the release of small and soluble molecules in the swollen process. ABS with higher swelling power has higher solubility than SBS and EBS at 95 °C. This trend was similar to that of babassu mesocarp starches [3]. Generally, the differences of swelling power and solubility of starches may be attributed to different extraction procedures, different granule structural properties and starch molecular weights [41].

### 2.7. Percent Transmittancy

**Figure 5:**
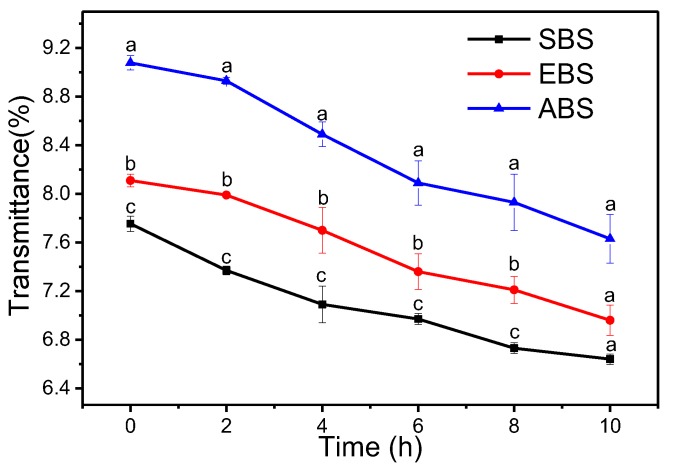
Paste clarity of SBS, EBS and ABS (Data were reported in means ± SD (*n* = 3); values in the same row with different superscripts are significantly different (*p* ˂ 0.05).

The clarity of starch gels was expressed as light transmittance by measuring the absorbance at 640 nm after fixed intervals of storage period. It is important in food and industrial applications as it affects product quality, in particular its aesthetic attractiveness. ABS presented greater clarity in comparison with EBS and SBS (Figure 5). The greater clarity of ABS can be attributed to the higher value of swelling power and its higher amylose content. Additionally, the low molecular weight, short chains of starch could also facilitate the higher light transmittance [22]. The changes in the granular and molecular structure induced by heating may lead to penetration and absorption of the starch granules, which ultimately results in the higher swelling of starch and thus a greater degree of light transmittance [23]. With the increase in storage time, the paste clarity of different starches decreased due to molecular realignment of solubilized starch chains [22]. 

### 2.8. Freeze-Thaw Stability

**Figure 6:**
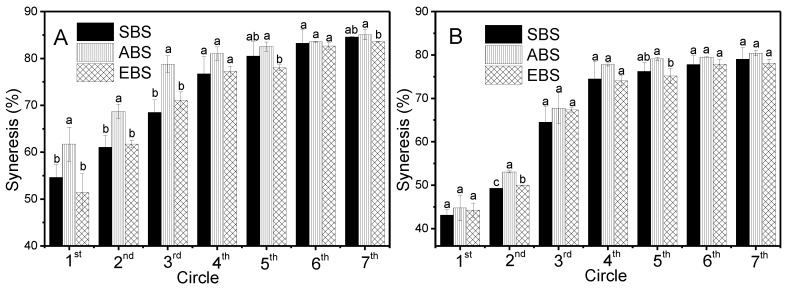
Syneresis of 3% starch (**A**) and 5% starch (**B**) (Each experiment was repeated three times and the number of samples was 3).

Freeze-thaw stability is an important property, which could be used to evaluate the ability of starch to withstand undesirable physical changes during freezing and thawing. The freeze-thaw stability during freeze-thaw cycles (1st, 2nd, 3rd, 4th, 5th, 6th and 7th) was evaluated with the percentage of syneresis and is exhibited in Figure 6.

A starch sample is freeze-thaw stable if it releases little or no exudate when subjected to freeze-thaw cycles. From the results of 3% and 5% starch samples (Figure 6), the syneresis increased rapidly in the first three cycles and then increased slowly in the next four cycles. This trend was similar to those of NPE, G50CE and G50PE syneresis values reported by Hong, et al [42]. Among the two concentrations of starch samples, ABS showed higher syneresis from 61.7% to 85.1% and 44.8% to 80.5%, respectively, and exhibited the poor freeze-thaw stability, which might be attributed to the higher amylose content (55.0%), polymerization degree of amylose, chain length of amylopectin, molecular size and so on [8]. However, SBS and EBS displayed lower syneresis and better freeze-thaw stability during the first three circles. This might be attributed to the similar amylopectin content of EBS and SBS, which might induce the large steric hindrance and help prevent water molecules separating out of the gel network due to the branches of amylopectin starch. Furthermore, there are significant differences in the syneresis values, 54.6% to 68.5% for SBS, 61.7% to 78.8% for ABS, and 51.4% to 71.1% for EBS in the first three circles of 3% starch samples. The syneresis of 5% starch was 43.1% to 64.5% for SBS, 44.8% to 67.4% for ABS, and 44.3% to 67.3% for EBS in the first three circles, respectively. However, the starch concentration effect on the syneresis property was unobvious after three circles. It was speculated that there was a steric hindrance effect of amylopectic starch on the syneresis property in the 5% starch sample, and this exact effect should be further verified.

### 2.9. Pasting Properties

**Figure 7:**
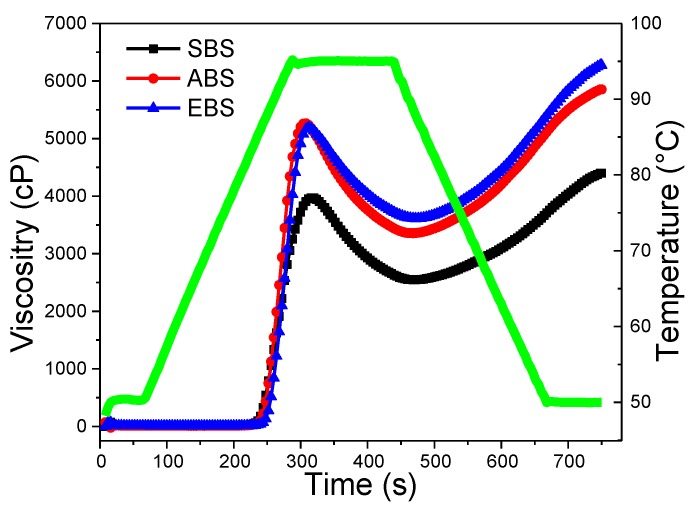
Pasting characteristics of SBS, EBS and ABS.

Pasting, which occurs after the gelatinization process, implicates granules swell in water to form a gel [43]. The pasting properties of starches obtained by different methods determined by a Rapid Visco Analyzer RVA at 160 rpm are presented in Figure 7 and Table 3. The peak viscosity was attributed to the water-holding capacity, while the lowest value of SBS in the peak viscosity might suggest poor water-holding capacity in the SBS system when compared to EBS and ABS [9]. More starch granules with a high swelling capacity result in a higher peak viscosity. The peak viscosity of ABS (5293 cP) was significantly higher than that of SBS, which might be due to the higher swelling power of ABS (18.6 g/g, at 95 °C) and amylose content, consisting of Cassava starches [8]. Peak viscosity could also be influenced by the protein composition differences, amylose leaching extent, amylose-lipid complex formation, friction between swollen granules, granule swelling and competition between leached amylose and remaining ungelatinised granules for free water [44]. All starches showed an increase in viscosity with the increasing temperature, which might be attributed to the removal of water from the starches granules as the temperature increased. The peak viscosity often correlates with the quality of the end-product, additionally providing an indication of the viscous load likely to be encountered by a mixing cooker. Higher peak viscosity in comparison with bulbil of Chinese yam, kidney bean starch, and traditional potato starches might be advantageous for their applications as a thickening agent in food systems [44]. However, ABS granules are also much more susceptible to disintegration, which results in high breakdown (1905 cP) and relatively low setback (2526 cP) values. Setback is defined as the degree of re-association between the starch molecules involving amylose [45]. Higher breakdown viscosity suggests the sample underwent a higher degree of swelling and subsequent disintegration, and the evidence is obtained from the highest swelling power of ABS (Figure 4). Sodium and magnesium correlated negatively with the breakdown.

Pasting temperature is a measure of the temperature at which a starch starts to thicken. The ability of starches to withstand heating at high temperature and shear stress is an important factor in many processes. EBS exhibited a higher pasting temperature (85.1 °C) than SBS (83.6 °C) and ABS (84.4 °C), in accordance with the gelatinization temperatures obtained by DSC (Table 2). The pasting temperature of 83.58 to 85.55 °C for bulbils starches observed in this study was similar to those of bulbils starches obtained by Zhou et al. [11], higher than that of yam (72.08 °C), rice (79.98 °C), potato (63.58 °C) and corn (82.08 °C), but lower than that of wheat (88.68 °C) starches [31]. SBS with low swelling power (14.1 g/g at 95 °C from Figure 4) has the lowest peak viscosity (3866 cP) and breakdown (1302 cP) and the lowest pasting temperature (83.6 °C) (Figure 4 and Table 3). Final viscosity (FV) indicates the ability of SBS, EBS and ABS to form a viscous paste range from 4290 cP to 6300 cP. The highest FV of EBS might be due to the aggregation of the amylose molecules.

## 3. Experimental

### 3.1. Materials and Reagents

**Figure 8:**
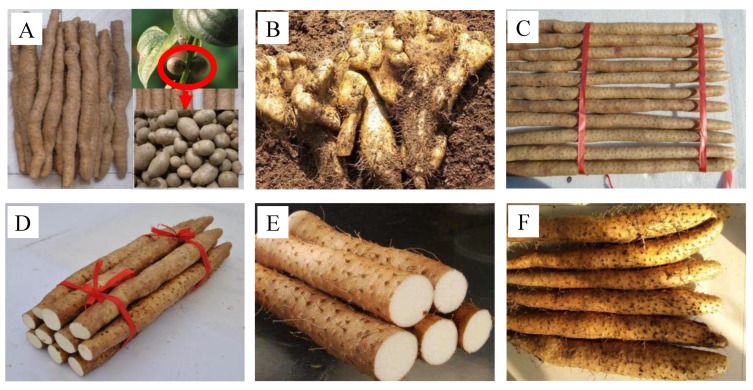
Photographs of six different *Dioscorea* plants. (**A**), *Dioscoreae opposita* Thunb. cv. Tiegun, bulbils were encircled by red circle; (**B**), Bergamot yam (Hubei Province, China); (**C**), *Dioscoreae opposita* Thunb. cv. Ma (Hebei Province, China); (**D**), *Dioscoreae opposita* Thunb. cv. Ximao (Shandong Province, China); (**E**), *Dioscoreae opposita* Thunb. cv. Jiujinhuang (Shandong Province, China); (**F**), *Dioscoreae opposita* Thunb. cv. Dahechangyu (Japanese).

Fresh bulbils of *Dioscorea opposite* Thunb. cv. Tiegun were collected from Wen County, Jiaozuo City, Henan Province of China. The morphological differences among *Dioscorea opposite* Thunb. cv. Tiegun and other varieties are visible in Figure 8. Alkaline proteinase was purchased from Solarbio company (Beijing, China). Unless otherwise stated, all chemicals used in the course of current work were of analytical grade.

### 3.2. Fractionation and Isolation of Starches

#### 3.2.1. Aqueous Steeping Method

The fresh bulbils were first washed with clean water and then pulped in a commercial kitchen blender (TM-767, Haipan electrical appliance Co., Ltd., Zhongshan City, China). The slurry was filtered using suction, and the large granules were retained and pulped again. The slurries were collected and washed with deionized water. After depositing, the supernatant was discarded and the upper non-white layer was physically scraped off. The remaining white layer was re-suspended in deionized water, followed by centrifugation at 4000 rpm for 15 min. The re-suspended procedure was repeated seven or eight times and the total duration was 10 h. The final starch product was dried in an oven (DHG-2640B, Zhengzhou Shengyuan Equipment Co. Ltd., Zhengzhou City, China) at 45 °C for 12 h. The dried starch was sieved using a 200-mesh sieve and collected and named as SBS. The yield was then calculated as the percentage of the fresh bulbils weight.

#### 3.2.2. Enzyme Extraction Method

The fresh bulbuls were washed, chopped into small pieces, then dried in an oven against spoilage at 45 °C for 24 h and stored in a desiccator at room temperature before use. Dried material of 100 g was soaked in 400 mL deionized water solution containing alkaline proteinase at a concentration of 100 mg at a pH of 10 (pH of the mixtures was adjusted using 0.1 M NaOH). The mixture was then incubated at 45 °C for 150 min and subsequently at 4 °C refrigerator for 3 h. The upper supernatant was discarded and the bottom starch layer was then pulped with a blender. The slurry was washed with deionized water and stirred for 3 h. After depositing, the upper layer was discarded and the upper non-white layer was physically scraped off. The remaining white layer was re-suspended in deionized water. The procedure was typically repeated seven or eight times until the upper supernatant turned clear and the starch layer exhibited a lightly white color appearance. The total duration was 10 h. The final starch product was dried in an oven at 45 °C for 12 h and then sieved with a 200-mesh sieve and collected and named as EBS. The yield was then calculated as the percentage of the dried bulbils weight.

#### 3.2.3. Alkaline Extraction Method

As described above, for sodium hydroxide extraction of starch, fresh bulbuls were first washed and chopped into small pieces and then dried against spoilage at 45 °C for 24 h and stored in a desiccator at room temperature before use. One-hundred grams of the dry materials was mixed with NaOH solution of 0.2% (*w*/*v*) concentration with a solid to liquid ratio of 1: 4, and the final pH was 13.80. The mixture was left at 35 °C for 3 h, then, in 4 °C refrigerator for 3 h. The top supernatant was discarded and the bottom starch layer was collected and pulped with a blender. A slurry product was obtained and then washed with deionized water, followed by stirring for 3 h. After depositing, the upper layer was discarded, and the upper non-white layer was physically scraped off. In addition, the remaining white layer was collected and re-suspended in deionized water and then subjected to stirring and depositing as described above. The procedure was repeated seven or eight times until the top supernatant layer turned clear and the starch layer exhibited a lightly white color; the total duration was 10 h. The final starch product was then dried at 45 °C for 12 h and sieved with a 200-mesh sieve and collected and named as ABS. The yield was calculated as the percentage of the dried bulbils weight.

### 3.3. Characterization

#### 3.3.1. Chemical Composition

The moisture, protein, lipid, phosphorus and ash contents of SBS, EBS and ABS were determined using the reported methods described by AOAC (2005) [46]. The amylose content was measured using the iodine colorimetric method [24]. All measurements were performed in triplicate. The mineral contents of three bulbils starch were determined according to the method described by Contreras-Jiménez et al. [17].

#### 3.3.2. Fourier Transform Infrared Spectroscopy (FT-IR)

FT-IR analyses of SBS, EBS and ABS were carried out on a Nicolet iN10 FT-IR spectrophotometer (Madison, WI, USA) equipped with a liquid nitrogen-cooled MCT detector. The spectra were recorded from 4000 to 650 cm^−1^ with a resolution of 4 cm^−1^.

The infrared crystalline index was calculated using the following formula:

(1) $$\mathrm{N-O}’\mathrm{KI}=\frac{\log(1/T_{1158})}{\log(1/T_{2931})}$$

where N-O’KI is the infrared crystal index, T_1158_ represents the transmittance of C–O–C stretching vibration absorption at 1158 cm^−1^, and T_2931_ indicates the transmittance of C–CH_2_–C stretching vibration absorption at 2931 cm^−1^.

#### 3.3.3. Scanning Electron Microscopy

The morphological characteristics of SBS, EBS and ABS were examined by scanning electron microscopy (SEM) (Hitachi S-3400N, Hitachi High-Tech Co., Tokyo, Japan). The samples were first coated with a thin gold layer and placed on the substrate. The corresponding images were then observed at a voltage of 5.0 kV with 500- and 1000-fold magnification under high vacuum.

#### 3.3.4. Differential Scanning Calorimetry (DSC)

The thermal behaviors of samples were measured using a differential scanning calorimeter Q100 (TA Instruments, USA) with nitrogen as purge gas. A starch sample of 3 mg was placed onto a DSC pan and 12 μL of distilled water was then added through a micro-syringe. An empty pan was used as a control and reference. The samples were scanned at a temperature range from 25 to 110 °C at a heating rate of 5 °C/min. The enthalpy variation (ΔH), onset temperature (To) and conclusion temperature (Tc) were calculated accordingly.

#### 3.3.5. X-ray Diffraction

The X-ray diffraction patterns of starch samples were collected using an X-ray diffractometer (Empyrean, PANalytical BV, Almelo, Netherlands) with Cu K_α_ radiation (λ=1.54 Å) and a linear focus at 40 kV and 15 mA. Measurements were performed in symmetric or powder geometry (*θ*-2θ) with a sweep from 2 to 40°, step size of 0.01 and rate of 3°/min [7]. 

#### 3.3.6. Swelling Power and Solubility

The swelling power and solubility of SBS, EBS and ABS were measured according to the method described by Leach et al. with slight modifications [38]. Samples of 0.1 g were weighted into centrifuge tubes and 10 mL of distilled water was then added. The suspensions were subsequently heated at temperatures of 55 °C, 65 °C, 75 °C, 85 °C, and 95 °C in a water bath for 30 min. The suspensions were then centrifuged at 8000 g for 20 min. To quantify the soluble fractions, the supernatants were removed and left to dry in an oven at 100 °C until constant weights were reached. The tubes containing the swollen starch granules were weighed for the determination of the swelling power. The solubility and swelling power of the samples were calculated using Equations (2) and (3), respectively:

(2) $$\mathrm{Solubility}\;(\%)=\frac{\mathrm{Weight}\;\mathrm{of}\;\mathrm{solid}\;\mathrm{solubles}\;(g)}{\mathrm{Weight}\;\mathrm{of}\;\mathrm{sample}\;(g)}\text{×}100$$

(3) $$\mathrm{Swelling}\;\mathrm{power}\;(g/g)=\frac{\mathrm{Weight}\;\mathrm{of}\;\mathrm{gel}\;(g)}{\mathrm{Weight}\;\mathrm{of}\;\mathrm{sample}\;(g)\;-\mathrm{Weight}\;\mathrm{of}\;\mathrm{solid}\;\mathrm{solubles}\;(g)\;}$$

#### 3.3.7. Starch Paste Clarity

Starch paste clarity was measured using the method reported by Craig et al [23]. Tubes containing starch suspensions (1%) with threaded caps were placed in boiling water for 30 min with vortexing every 5 min, and then let to cool to room temperature. The percentage of transmittances (%T) of the samples at a wavelength of 650 nm was determined using a UV1901PC spectrophotometer (Shanghai Yoke Instrument Co., Ltd., Shanghai, China). 

#### 3.3.8. Freeze-Thaw Stability

Freeze-thaw stability was investigated using a method reported by Hoover et al. [47] with a slight modification. Briefly, 400 mL of 3% and 5% starch suspension was heated to 95 °C at a rate of 5 °C/min and kept at this temperature for 15 min, then cooled to and kept at 50 °C at the same rate for 15 min. Fifty millilters of the treated samples were transferred to centrifuge tubes and left to cool down to room temperature. The samples were then frozen at −20 °C for 24 h. For the measurement of freeze-thaw stability, the samples were thawed at 25 °C for 4 h, micro-centrifuged at 4000 rpm for 10 min and the water separated from the starch gel was recorded. Seven freeze-thaw cycles were performed as described above. The percentage of water separated after each freeze-thaw cycle was measured and the data were reported as the average of the three measurements. The syneresis rate was calculated with Equation (4).

(4) $$\mathrm{Syneresis}\;\%=\frac{\mathrm{Weight}\;\mathrm{of}\;\mathrm{syneresis}\;\mathrm{water}\;(g)}{\mathrm{Weight}\;\mathrm{of}\;\mathrm{gel}\;(g)}\times 100$$

#### 3.3.9. Pasting Properties

Pasting properties of three different bulbil starches were evaluated using Rapid Visco Analyzer (RVA) (Techmaster, Perten Instruments, Sweden). Dry starches were mixed with distilled water to a concentration of 10% (*w*/*w*). The mixture was stirred at 960 rpm for 10 s until a state of homogenous dispersion was reached. The sample was then subjected to rotation at 160 rpm. Samples were kept at 50 °C for 1 min, then heated from 50 °C to 95 °C at a rate of 12 °C/min, kept at 95 °C for 2.5 min, followed by cooling from 95 °C to 50 °C at a rate of 12 °C/min and holding at 50 °C for 0.5 min. The total analysis time was 12.5 min. Peak viscosity, trough, breakdown, final viscosity, setback, and pasting temperature were recorded [21].

## 4. Conclusions

The bulbils starches isolated by three methods in this work presented distinctive chemical, mineral composition, and functional properties, which indicated that they could be the promising alternatives in food and other industries. Starch isolated from bulbils of *Dioscoreae opposita* Thunb. cv. Tiegun by the aqueous steeping extraction method afforded higher extraction yield and lower starch content as compared with enzyme and alkaline extraction methods. The starch granules were oval, spherical and kidney-shaped and the surface of the ABS and SBS granules appeared to be slightly rough. The starches having larger average granule size showed more amylose and phosphorus contents than those with smaller average granule size. The FT-IR spectra obtained for the three starches were similar in the form of the major peaks. Additionally, the ABS showed a higher gelatinization temperature (67.9 °C) indicating the higher stability of the starch crystallites and more heating was required to swell the granules, which could be a potential alternative during the cooking process in the industries. SBS and EBS displayed lower syneresis and better freeze-thaw stability in contrast to the corresponding ABS during the first three circles. These characteristics made SBS and EBS potential technological alternatives in quality preservation of frozen starch-based products and other industrial applications. Furthermore, ABS and EBS had the higher peak viscosities, final viscosities, setbacks and were suitable for use as food thickeners or frozen food additives. ABS had the best transparency and could be used for making medicine.

In conclusion, different isolation methods induced changes in the morphology of starch granules and chemical composition, which led to distinct functional properties. In the practical application process, people could choose the proper bulbil starch obtained by different extraction methods according to different characteristics.

## Figures and Tables

**Table 1:** Compositions and yields of starches.

Parameter	SBS	EBS	ABS
Yield (%)	29.9 ± 0.54a	25.5 ± 0.78b	23.8 ± 0.21c
Moisture content (%)	5.5 ± 0.10b	7.4 ± 0.08a	8.1 ± 0.02a
Protein (%)	0.01 ± 0.00a	0.01 ± 0.00a	0.01 ± 0.00a
Ash (%)	0.24 ± 0.00a	0.18 ± 0.02a	0.14 ± 0.03a
Lipids (%)	0.84 ± 0.18a	0.72 ± 0.11a	0.71 ± 0.25a
Starch (%)	88.9 ± 1.84b	89.6 ± 0.32ab	91.7 ± 0.03a
Amylose (%)	50.2 ± 1.09b	49.2 ± 0.17b	55.0 ± 0.43a
Mineral elements (mg/kg)			
P	127 ± 4.61b	160 ± 6.29a	174 ± 16.32a
B	1.13 ± 0.01a	1.11 ± 0.04a	1.08 ± 0.06a
Na	30.39 ± 20.07b	58.06 ± 13.07a	4.08 ± 0.001c
Mg	87.65 ± 23.82a	96.08 ± 16.6a	39.4 ± 17.88b
K	-	-	-
Ca	404.22 ± 85.49ab	464.01 ± 83.71a	255.93 ± 88.42c
Fe	-	17.25 ± 0.2	-
Cu	0.7 ± 0.01b	1.32 ± 0.09a	0.18 ± 0.004c
Zn	12.88 ± 0.27b	10.49 ± 0.15c	55.59 ± 0.75a
Se	0.06 ± 0.008b	0.21 ± 0.08a	0.04 ± 0.02b

Data were reported in means ± SD (*n* = 3); values in the same row with different superscripts are significantly different (*p* ˂ 0.05); “–”means not detected.

**Table 2:** Gelatinization parameters of starches (SBS, EBS and ABS).

Parameter	SBS	ABS	EBS
*T_o_* (°C)	65.8 ± 0.2c	67.9 ± 0.3a	66.8 ± 0.1b
*T_p_* (°C)	70.3 ± 0.3b	69.3 ± 0.2c	73.0 ± 0.4a
*T_c_* (°C)	76.3 ± 0.3b	73.0 ± 0.1c	82.0 ± 0.2a
ΔH_gel_ (J/g)	2.0 ± 0.1c	13.8 ± 0.1a	11.5 ± 0.3b
(*T_c_*−*T_o_*) (°C)	10.5 ± 0.4b	5.1 ± 0.3c	15.2 ± 0.5a

*T_o_*, onset gelatinization temperature; *T_p_*, peak gelatinization temperature; *T_c_*, conclusion gelatinization temperature; (*T_c_*−*T_o_*), gelatinization range; ΔHgel, gelatinization enthalpy. All data represent the mean of triplicates. Values in each column with different superscripts are significantly different (*p* < 0.05).

**Table 3:** Pasting properties of SBS, EBS and ABS.

Sample	Peak Viscosity (cP)	Trough (cP)	Breakdown (cP)	Final Viscosity (cP)	Setback (cP)	Pasting Temperature (°C)
SBS	3919 ± 53.0c	2556 ± 8.5c	1364 ± 61.5c	4344 ± 53.5c	1788 ± 62c	83.58 ± 0.03c
EBS	5192 ± 2.5b	3626 ± 9.5a	1571 ± 12b	6287 ± 13.5a	2666 ± 4a	85.55 ± 0.45a
ABS	5293 ± 30.0a	3388 ± 32.0b	1905 ± 2a	5914 ± 61.5b	2526 ± 29.5b	84.35 ± 0.05b

Data were reported in means ± SD (*n* = 3); Values in the same row with different superscripts are significantly different (*p* ˂ 0.05).
